# Delirium in Elderly Hospitalized Patients: Analyzing Clinical and Social Determinants in a Colombian Retrospective Cohort

**DOI:** 10.1002/gps.70079

**Published:** 2025-04-20

**Authors:** Alejandra Ospina‐Lehmann, María Camila Castañeda‐Gómez, Eduardo José Pabón‐Martínez, Juan Pablo Vigoya‐Aponte, Santiago Orozco‐Castro, Diego Andrés Chavarro‐Carvajal, Carlos Alberto Cano‐Gutiérrez

**Affiliations:** ^1^ Semillero de Neurociencias y Envejecimiento Aging Institute Medical School Pontificia Universidad Javeriana Bogotá Colombia; ^2^ Clinical Epidemiology and Biostatistics Department Medical School Pontificia Universidad Javeriana Bogotá Colombia; ^3^ Geriatrics Unit Hospital Universitario San Ignacio Bogotá Colombia

**Keywords:** aged, delirium, prevalence, social determinants of health, sociodemographic factors

## Abstract

**Objective:**

This study aims to describe the clinical and social determinants associated with delirium in elderly patients hospitalized in the geriatrics department of Hospital Universitario San Ignacio (HUSI), in Bogotá, Colombia, between June 2019 and June 2022.

**Methods:**

We conducted a retrospective analytical study. Data were extracted from the hospital's electronic medical records. The primary outcome was delirium upon admission. Exposure variables included clinical (e.g., malnutrition, dementia, oropharyngeal dysphagia) and social factors (e.g., living alone, social networks). Associations between delirium and exposure variables were assessed using a multivariate logistic regression model.

**Results:**

The studied cohort comprised 4601 patients, mean age of 83.93 years and 56.26% were women. The prevalence of delirium upon admission was 22.39%. Key factors associated with delirium included older age (OR 1.04, 95% CI 1.01–1.06), malnutrition (OR 2.42, 95% CI 1.93–2.79), dementia (OR 2.57, 95% CI 2.02–3.38), functional impairment (OR 2.50, 95% CI 1.74–3.59), and oropharyngeal dysphagia (OR 1.49, 95% CI 1.08–1.99). Social determinants such as female sex, limited social networks, living alone, and enrollment in the subsidized health regime were not significantly associated with delirium.

**Conclusion:**

Delirium upon admission is highly prevalent among elderly inpatients and is associated with clinical factors, particularly malnutrition, dementia, and oropharyngeal dysphagia. Although no significant associations were found between social determinants of health (SDH) and delirium in this cohort, further research in diverse healthcare settings is needed to better understand the broader impact of SDH on delirium risk.

**Summary**

- The prevalence of delirium among elderly patients hospitalized in the geriatrics department was 22.39%. Delirium was associated with various geriatric syndromes, including malnutrition, dementia, functional impairment, and oropharyngeal dysphagia.
- Contrary to expectations, social determinants such as living alone, limited social networks, and enrollment in the subsidized health regime were not significantly associated with delirium risk in this cohort.
- Further research is needed to examine the relationship between delirium and other social determinants, such as ethnicity, poverty‐related factors (e.g., income or socioeconomic status), educational attainment, and employment status.

## 1 Introduction

Delirium is an acute neuropsychiatric disorder that affects attention, orientation, and awareness. It can also impair language, motor behavior, and sleep‐wake cycles [1]. It is considered a medical emergency due to its association with adverse outcomes, including increased morbidity, mortality, and prolonged hospital stays [2]. The etiology is often multifactorial, resulting from underlying conditions such as metabolic imbalances, sepsis, severe trauma, or cardiopulmonary diseases [2, 3]. This aligns with the fifth criterion of the DSM‐V for delirium: “the disturbance is a direct physiological consequence of another medical condition” [4].

Delirium is more common in the elderly, affecting 20% of the 12.5 million hospitalized individuals over 65 in the U.S. [5]. This increased prevalence is due to age‐related declines in the body's ability to regulate metabolic disruptions that affect brain function. In Colombia, between 2014 and 2020, 5104 cases of acute confusional syndrome were reported, with 55% occurring in females [6] and 88% from the elderly population [7]. During this period, 1021 deaths occurred in intensive care unit (ICU) patients with delirium, 94% of whom were over 60 [8]. The disability‐adjusted life years (DALYs) lost totaled 11,065, with 82% due to years of life lost and 18% due to years lived with disability, with Bogotá D.C. reporting the highest numbers [6].

In addition to predisposing medical factors, social determinants of health (SDH), defined by the World Health Organization (WHO) as “the conditions in which people are born, grow, work, live, and age” [9], significantly impact health outcomes. These determinants arise from the distribution of money, power, and resources at national and global levels and are key drivers of health inequities [10]. In elderly patients, SDH can be categorized into individual factors, social participation, and environmental characteristics. A strong support network, higher education, better socioeconomic status, and living in a high‐income country are associated with healthier aging [11].

In older adults with delirium, SDH play a crucial role in the onset of the condition during hospital admission. The cognitive reserve model suggests that factors such as education, socioeconomic status, and parental occupation throughout life can increase brain vulnerability and lower cognitive reserve [12, 13]. Additionally, SDH affect healthcare access, as socioeconomic status and geographic location can create barriers to timely care [12]. Social biases related to race, gender, or sexual orientation may also contribute to disparities in treatment, leading to longer wait times, misdiagnosis, and inadequate care, particularly among minority groups [13, 14].

This study addresses a research gap as being the first to investigate the role of social determinants in delirium among elderly patients in Colombia. Unlike previous studies conducted in high‐income countries [15], this research focuses on patients hospitalized in the geriatrics department of Hospital Universitario San Ignacio (HUSI) between June 2019 and June 2022. By examining the social and clinical factors contributing to delirium, this study aims to identify vulnerable populations, reduce health disparities, and develop effective preventive, diagnostic, and therapeutic strategies.

## 2 Materials and Methods

We performed an analytical retrospective study involving a cohort of patients hospitalized in the acute care unit of the Geriatrics department at HUSI between June 2019 and December 2022.

HUSI is a high‐complexity university hospital located in Bogotá, Colombia. Its Geriatrics Unit, a pioneer in patient‐centered care for older adults in the country, has 60 hospitalization beds and is staffed by five geriatricians. This unit provides specialized inpatient medical care for patients with acute conditions or exacerbations of chronic diseases, with a primary focus on individuals aged 75 years or older, as well as those aged 65 years or older presenting with highly complex conditions. These conditions include chronic disabling diseases, multimorbidity, cognitive impairments, geriatric syndromes, polypharmacy, frequent hospital readmissions, and significant social challenges.

Patient identification was based on hospitalization records from the geriatrics service and the hospital's electronic medical record system (SAHI). A trained data entry specialist was responsible for reviewing the medical records and entering the data into the REDCap platform for analysis.

Patients were included if they met the admission criteria for the HUSI geriatric acute care unit. Patients who had been referred to another institution, those hospitalized for 24 h or less, those with incomplete data, or those who developed delirium during hospitalization were excluded. The sampling was non‐probabilistic, with all patients intentionally selected from the database. As a retrospective intrahospital study, no follow‐up was required, and no sample adjustments were necessary. Scales and indices were collected systematically for each patient following the institutional protocol.

### 2.1 Variables

#### 2.1.1 Outcome Variable

The outcome variable, delirium, was assessed using the Confusion Assessment Method (CAM) at the time of admission [16]. Delirium was considered present if at least three of the four CAM criteria were met. The screening tool was routinely applied to all patients admitted to the geriatrics service during the study period.

#### 2.1.2 Exposure Variables

##### 2.1.2.1 Sociodemographic Variables

The exposure variables analyzed included age (measured in years), sex, and the patient's health insurance regime. In Colombia, the health insurance system is part of the country's Universal Health Coverage framework, and is divided into two main regimes. The contributory regime covers individuals with financial or labor capacity; and the subsidized regime provides essential healthcare services to low‐income and vulnerable populations, funded by government allocations and cross‐subsidies from the contributory regimen. This structure aims to reduce healthcare disparities and promote equitable access to medical services across socioeconomic groups.

##### 2.1.2.2 Functional Assessment Variables

Functional impairment was assessed using the Barthel Index for Activities of Daily Living (ADL) [17], with scores below 90 indicating impairment and scores ≥ 90 indicating full independence. All assessment scales were administered according to the institutional protocol and were systematically recorded in the admission clinical records of the acute geriatric care unit. This process was uniformly applied to all patients admitted to the unit, ensuring consistency in data collection. A family member or caregiver was always present to provide baseline information and assist in completing the assessment scales.

##### 2.1.2.3 Clinical Variables

The number of comorbidities, number of falls prior to admission in the last year and number of hospitalizations were explored during the admission and used as a continuous variable. Nutritional status was evaluated using the Mini Nutritional Assessment Short Form (MNA‐SF) [18], categorizing participants into three groups: normal nutritional status (12–14 points), risk of malnutrition (8–11 points) and malnutrition (7 points or lower). Oropharyngeal dysphagia was assessed through positive responses to standardized screening questions outlined in the institutional protocol interview [19].

##### 2.1.2.4 Mental Variables

A history of dementia was recorded based on reports obtained during the admission interview, including a prior diagnosis of dementia or documentation of ongoing treatment by a specialist in neurology, psychiatry, or geriatrics.

##### 2.1.2.5 Social Variables

Living alone and social networks were assessed via a semi‐structured interview conducted at the time of admission, in accordance with institutional protocol [19]. Social risk was defined by the patient's perception of their support network. Patients were classified into three categories: strong social network, social risk, and poor social network. This classification was later used as a dichotomous variable, distinguishing between strong or limited social networks for assessing social risk and poor social networks.

### 2.2 Statistical Analysis

A descriptive analysis was conducted on the variables of interest, using appropriate measures of central tendency and dispersion. Means, medians and standard deviations (SD) were reported depending on the normality or non‐normality of the distribution, which was assessed with the Shapiro–Wilk test. Categorical variables (women, subsidized healthcare plan, functional impairment, malnutrition, oropharyngeal dysphagia, dementia, limited social network, living alone) are presented as frequency tables, reporting both the number of events and percentage values. For non‐parametric continuous variables (age in years, average number of comorbidities, average number of falls, average number of hospitalizations), the median and SD were used as summary measures.

Bivariate models were employed to identify associations with delirium, which was initially analyzed as a dichotomous variable. Chi‐square tests were performed to assess relationships with categorical variables, while the Mann–Whitney *U* test was applied for continuous variables with non‐normal distribution. To control potential confounding factors, a multivariate regression model was subsequently developed, adjusting for variables such as sex, age, and independent factors. The model reported odds ratios (OR) and 95% confidence intervals (CI), both unadjusted and adjusted for confounders. Statistical significance was defined as *p* < 0.05 and all analyses were performed out using STATA 16.1.

## 3 Results

A total of 4601 patients were analyzed, with a mean age of 83.93 years; 56.26% were female, and 4.12% were enrolled in the subsidized health regime. The prevalence of delirium upon admission was 22.39%.

Regarding the baseline characteristics, 5.97% of patients lived alone, 8.04% had a limited social network, and 22.16% were malnourished. Additionally, 70.04% of the patients demonstrated dependence in basic activities, 54.11% had a diagnosis of dementia, and 9.9% presented with oropharyngeal dysphagia. The average number of reported falls was 0.43 (SD 1.38), while the average number of comorbidities was 2.08 (SD 1.31), and the average number of hospitalizations was 0.44 (SD 0.89) (Table 1).

**TABLE 1:** Baseline characteristics according to the presence or absence of delirium during hospitalization.

Variable	Total	Delirium (yes)	Delirium (no)	*p*
	*n* = 4601	*n* = 1030 (22.39%)	*n* = 3571 (56.26%)	
Sociodemographic variables				
Age in years, mean (SD)	83.93 (5.22)	85.27 (5.82)	83.54 (4.97)	< 0.001
Female, *n* (%)	2588 (56.26%)	605 (58.74%)	1587 (44.45%)	0.069
Subsidized healthcare plan, *n* (%)	199 (4.12%)	43 (4.17%)	156 (4.38%)	0.777
Functional assessment variables				
Functional impairment, *n* (%)	3223 (70.04%)	894 (88.78%)	2309 (66.6%)	< 0.001
Clinical variables				
Average number of comorbidities, mean (SD)	2.08 (1.31)	2.08 (1.30)	2.08 (1.31)	0.924
Average number of falls, mean (SD)	0.43 (1.38)	0.58 (1.22)	0.39 (1.03)	0.001
Average number of hospitalizations, mean (SD)	0.44 (0.89)	0.52 (0.89)	0.42 (0.89)	0.001
Malnutrition, *n* (%)	1020 (22.16%)	395 (38.49%)	625 (17.50%)	< 0.001
Oropharyngeal dysphagia, *n* (%)	456 (9.901%)	162 (15.72%)	294 (8.93%)	< 0.001
Mental variables				
Dementia, *n* (%)	2490 (54.11%)	795 (77.18%)	1695 (47.47%)	< 0.001
Social variables				
Limited social network, *n* (%)	370 (8.04%)	88 (8.54%)	282 (7.89%)	0.551
Living alone, *n* (%)	275 (5.97%)	47 (4.56%)	228 (6.38%)	0.018

The analysis of the population characteristics is presented in Table 1. Compared to patients without delirium, those with delirium were older (85.27 years [SD 5.82] vs. 83.54 years [SD 4.97], *p* < 0.001), had a higher number of falls (0.58 [SD 1.22] vs. 0.39 [SD 1.03], *p* = 0.001), and more hospitalizations (0.52 [SD 0.89] vs. 0.42 [SD 0.89], *p* = 0.001). Furthermore, they also exhibited higher rates of malnutrition (38.49% vs. 17.50%, *p* < 0.001), oropharyngeal dysphagia (15.72% vs. 8.93%, *p* < 0.001), dementia (77.18% vs. 47.47%, *p* < 0.001) and functional impairment (88.78% vs. 66.6%, *p* < 0.001).

In the multivariate logistic regression analysis (Table 2), risk factors associated with delirium upon admission included older age (OR 1.04, CI 95% 1.01–1.06, *p* = 0.001), functional impairment (OR 2.50, CI 95% 1.74–3.59, *p* < 0.001), malnutrition (OR 2.42, CI 95% 1.93–2.79, *p* < 0.001), dementia (OR 2.57, CI 95% 2.02–3.38, *p* < 0.001) and oropharyngeal dysphagia (OR 1.49, CI 95% 1.08–1.99, *p* = 0.029). Conversely, SDH such as being female (OR 0.93, CI 95% 0.75–1.17, *p* = 0.576), enrollment in the subsidized healthcare plan (OR 1.04, CI 95% 0.6–1.79, *p* = 0.874), having a limited social network (OR 0.87, CI 95% 0.6–1.26, *p* = 0.478), and living alone (OR 1.22, CI 95% 0.73–2.05, *p* = 0.434), were not significantly associated with delirium.

**TABLE 2:** Factors associated with delirium prevalence during hospitalization: A multivariate analysis.

Variable	Crude OR		Adjusted OR^a^	
	OR (CI 95%)	*p*	OR (CI 95%)	*p*
Sociodemographic variables				
Age in years	1.06 (1.05–1.07)	< 0.001	1.04 (1.01–1.06)	0.001
Female	1.13 (0.98–1.31)	0.069	0.93 (0.75–1.17)	0.576
Subsidized healthcare plan	0.95 (0.67–1.34)	0.777	1.04 (0.60–1.79)	0.874
Functional assessment variables				
Functional impairment	3.96 (3.22–4.88)	< 0.001	2.50 (1.74–3.59)	< 0.001
Clinical variables				
Average number of comorbidities	1.07 (0.84–1.36)	0.554	0.98 (0.90–1.06)	0.752
Average number of falls	1.14 (1.06–1.23)	< 0.001	1.06 (0.97–1.15)	0.193
Average number of hospitalizations	1.12 (1.04–1.20)	0.002	1.00 (0.91–1.12)	0.193
Malnutrition	3.18 (2.72–3.73)	< 0.001	2.42 (1.93–2.79)	< 0.001
Oropharyngeal dysphagia	2.10 (1.69–2.60)	< 0.001	1.49 (1.08–1.99)	0.014
Mental variables				
Dementia	3.74 (3.19–4.39)	< 0.001	2.57 (2.02–3.38)	< 0.001
Social variables				
Limited social network	1.07 (0.83–13.8)	0.551	0.87 (0.60–1.26)	0.478
Living alone	0.67 (0.49–0.93)	0.019	1.22 (0.73–2.05)	0.434

^a^ Adjusted for: sex, age, and independent factors.

## 4 Discussion

The present study provides valuable insights into the morbidity profile of older adult patients with delirium admitted to a high‐complexity hospital in Bogotá, Colombia, and analyzes the clinical and social determinants associated with this syndrome. Our findings reveal that delirium is a prevalent condition among patients hospitalized in the acute care unit, with a prevalence of 22.39%. Additionally, we observed a high burden of geriatric syndromes, such as functional dependency, falls, malnutrition, and cognitive decline, which are consistent with findings from other cohorts [20, 21]. This pattern suggests a heightened vulnerability among this patient population and highlights the importance of a comprehensive geriatric assessment in the management of older adults with delirium.

Our results are consistent with those reported in other studies. For instance, a study in Italy reported a prevalence of 22.9% in older patients [20], while a British cohort observed a prevalence of 27% [21]. In the Latin American context, a systematic review indicated that the prevalence of delirium upon hospital admission ranges from 10% to 31% [22]. Comparatively, our prevalence is slightly higher than that found in Argentina (18.6%) [23] but lower than in Chile (35.4%) [24]. Furthermore, when comparing our current data with those from 2017 (51.03%) [25], we observe a notable decrease in the prevalence of delirium. This decline may be attributed to an increase in the number of patients admitted to our acute care unit, as well as improvements in the detection of at‐risk patients and the implementation of anti‐delirium preventive measures according to institutional protocol.

Our study further highlights the significant association between delirium upon admission and geriatric syndromes. Notably, malnutrition has been recognized in the literature as a key risk factor for delirium. As demonstrated in previous systematic reviews and meta‐analyses [26], malnutrition not only contributes to cognitive decline but also serves as a modifiable risk factor. Addressing and treating malnutrition could potentially contribute to the resolution of delirium [27]. Therefore, the importance of early identification and intervention in the treatment of malnutrition in older adults at risk for delirium is demonstrated.

Oropharyngeal dysphagia also emerged as an independent factor associated with delirium, aligning with previous studies [28]. Dysphagia likely contributes to delirium through multiple mechanisms, including gastrointestinal dysfunction, inflammatory responses, and disruption of neurotransmitter balance [29]. These results enhance the need for systematic screening for dysphagia in institutional protocols to optimize the management of patients with delirium.

Moreover, other geriatric syndromes, such as functional impairment and dementia, were identified as independent factors associated with delirium, in agreement with previous research [20]. The high prevalence of dementia in our cohort is particularly striking and aligns with findings from prior studies conducted in our unit [30]. This may be explained by the presence of a Memory and Cognition Center at HUSI, where the management of acute conditions in these patients is often carried out within our acute geriatric unit.

In accordance with Inouye's multifactorial model [31], our findings highlight the role of predisposing factors (including advanced age, functional limitations, and cognitive decline) in increasing susceptibility to delirium. These results underscore the complex and multifactorial nature of delirium, emphasizing the relevance of a holistic, interdisciplinary approach to its management in older adults.

In the present study, no significant association was found between social determinants and delirium. While the absence of private health insurance has been described in the literature as an independent factor associated with delirium in other cohorts [32], this trend was not observed in our population. In Colombia, the subsidized health regime guarantees basic and free access to healthcare services, including hospital care and comprehensive management, which might mitigate some risk factors for delirium, such as delayed medical attention, limited access to essential medications, and disruptions in continuity of care. In contrast, in other countries, the absence of private insurance may be linked to greater economic and logistical barriers to accessing high‐quality healthcare services. It is worth noting that only 4.12% of the patients in our cohort were affiliated with the subsidized system, which is not representative of the national population. Future studies are necessary to further explore this variable and its impact on delirium in diverse healthcare settings.

Other determinants, such as living alone or being female, were not associated with delirium upon admission in our study, which is consistent with findings reported in other studies [32]. This could be explained in our population by factors such as the development of informal support networks among older adults and the higher healthcare utilization rates among women compared to men [33].

Although we were unable to find evidence from one Colombian region indicating that SDH influenced the onset of delirium upon admission, this does not preclude the existence of such relationships in other populations within our country or in other regions [34]. Moreover, due to limitations in data collection, we were unable to include other potentially relevant variables, such as ethnicity, poverty‐related indicators (e.g., income or socioeconomic status), educational level, and employment status. Future research should study the relationship between delirium and these variables in our population, as well as in other countries with similar sociocultural and healthcare contexts.

The strengths of our study include its robust sample size and its distinction as the first to introduce the concept of SDH in the evaluation of delirium upon admission among older adults in our region. Additionally, the study employed a systematic assessment protocol aligned with a multidimensional approach, utilizing validated screening scales administered by trained personnel. Nevertheless, our study also has limitations. First, the retrospective design and reliance on secondary analysis of pre‐existing medical records introduce potential biases due to incomplete or inconsistent data, as the information was not specifically collected for the purposes of this study. Second, the use of non‐probabilistic sampling may have introduced selection bias, potentially limiting the representativeness of the findings. Third, conducting the study within a single healthcare institution restricts the external validity of the results, thereby limiting their generalizability to other healthcare settings or populations. Finally, the study did not account for admission etiologies, which may introduce confounding variables. Conditions such as infections, metabolic imbalances, or neurological disorders could influence both delirium prevalence and its association with social determinants of health. The absence of this variable limits the control of potential interactions between clinical and social factors, increasing the risk of residual confounding. Future research should integrate admission diagnoses to strengthen the validity of these findings.

## 5 Conclusion

This study highlights a significant prevalence of delirium upon admission among hospitalized elderly patients, with 22% of the patients exhibiting this condition. Key risk factors, such as malnutrition, dementia, and oropharyngeal dysphagia, were significantly associated with delirium. Although our study does not suggest an association between delirium and certain SDH, further research within the Colombian context is needed to better understand this potential relationship. Such insights could inform policymakers in developing public health strategies for the prevention and management of delirium in older adults.

## Ethics Statement

The study received approval from the Institutional Research and Ethics Committee of the Faculty of Medicine of the Pontificia Universidad Javeriana and the San Ignacio University Hospital, under registration number 14/2023. This approval confirms our full adherence to the ethical and scientific principles outlined in the Declaration of Helsinki, 64th General Assembly, Fortaleza, Brazil, October 2013; Resolution 8430 of 1993, which establishes the scientific, technical, and administrative standards for health research; the Guidelines for Harmonisation, Good Clinical Practice in Research, International Council for Harmonisation of Technical Requirements for Pharmaceuticals for Human Use (ICH); and Resolution 2378 of 2008, which adopts Good Clinical Practices for institutions conducting research.

## Conflicts of Interest

The authors declare no conflicts of interest.

## Data Availability

The data that support the findings of this study are available from the corresponding author upon reasonable request.
